# Editorial: Health professions education at a time of triple planetary crises

**DOI:** 10.3389/fmed.2025.1687379

**Published:** 2025-11-04

**Authors:** Komal Atta, Fathima Rizka Ihsan, Jacqueline G. Bloomfield, Lynn V. Monrouxe, Michelle McLean

**Affiliations:** ^1^Department of Medical Education, University Medical and Dental College, The University of Faisalabad, Faisalabad, Pakistan; ^2^Faculty of Medicine and Health, Sydney School of Health Sciences, The University of Sydney, Camperdown, NSW, Australia; ^3^Faculty of Medicine and Health, Sydney Nursing School, The University of Sydney, Camperdown, NSW, Australia; ^4^Faculty of Health Sciences & Medicine, Bond University, Gold Coast, QLD, Australia

**Keywords:** planetary health, climate change, health profession education, triple planetary crisis, planetary health collaborations

This Research Topic, “*Health professions education at a time of triple planetary crises*,” includes nine articles that explore various dimensions of how health professions education must respond to the interrelated crises of a changing climate, biodiversity loss, and pollution. Collectively referred to as a “Triple Planetary Crisis,” this phrase is no longer metaphorical. In late 2023, editors of more than 200 health journals urged the World Health Organization (WHO) to declare this interconnected climate-nature crisis a global health emergency, warning that it meets all the criteria for severity, international impact, and urgency of action (1). Separating the climate and nature (biodiversity loss and pollution) crises has led to fragmented responses and missed opportunities in terms of synergistic solutions. It is important to understand that the Planetary Health crisis encompasses climate change, biodiversity loss and pollution and needs to be addressed urgently. Subsequently, the development of eco-ethical leadership for dealing with planetary changes is an imperative in healthcare professions education (2).

The articles in this Research Topic weave together three interconnected threads that form the fabric of Planetary Health education: professional involvement, curriculum development, and student agency. Each theme strengthens and supports the others, creating a robust framework for transformative learning.

Professional involvement develops as healthcare practitioners confront their expanding role in addressing environmental challenges. The evolving perspectives of young doctors on climate-related issues (Segala et al.) illuminate this professional awakening, while the cultivation of Planetary Health literacy (Okatch et al.) equips practitioners with essential knowledge. Most urgently, the recognition that health professionals must address the triple planetary catastrophe (1) positions the medical community as active agents of change rather than passive observers.

Curriculum development serves as the structural foundation, providing the educational architecture needed to embed these critical concepts into formal learning pathways. This systematic integration ensures that Planetary Health education moves beyond *ad hoc* initiatives to become a core component of professional training (Pais Rodrigues et al.).

Student agency acts as the dynamic catalyst that brings theory into practice. Through the co-creation of learning resources (Pais Rodrigues et al.), students evolve from passive recipients to active architects of their education. This empowerment extends to learner-driven innovation, which is supported by the implementation of school kits (Lokmic-Tomkins et al.) and enriched by interdisciplinary approaches (Grieco et al.). Recognizing that engagement requires emotional and intellectual support, techniques for promoting student wellbeing help learners navigate the anxiety that can accompany confronting planetary-scale challenges (Malmqvist and Oudin).

Together, these three themes create a synergistic approach in which professional responsibility drives curricular innovation, student agency shapes meaningful learning experiences, and curriculum development provides the framework for sustainable change. Indeed, healthcare professionals have historically played a vital role in advocating for public health issues and they must now extend this advocacy to the triple planetary crisis, especially considering the health sector's own environmental footprint (Malmqvist and Oudin). Policymakers and educators must create a well-rounded, trickle-down policy on Planetary Health literacy, which includes educating the public. Underlying the scholarly contributions in this Research Topic is a foundational recognition—the triple planetary crisis is a health crisis. These articles highlight that including Planetary Health is not an optional extra in health professions education. It is a necessity to prepare knowledgeable health professionals who are skilled to take action to address our climate-nature crisis.

First, while there is consensus on the necessity, even the ethical obligation, of integrating Planetary Health into the education of healthcare professionals (Lokmic-Tomkins et al.) to prepare graduates to address the direct and indirect impacts of environmental degradation, implementation gaps persist. To this end, for Grieco et al., although the majority of German medical schools offer Planetary Health content, it is mainly electives, with little “core” curriculum. Segala et al.'s research found that although young Italian doctors and medical students exhibit substantial climate anxiety and are willing to act, their formal education about climate-health connections remains minimal. Similarly, while not a part of this Research Topic, studies in the Arab and Asian contexts show that despite awareness of the health threats of the triple crisis, policies and programs for action are scarce (3). Collectively, these studies suggest that while awareness is growing, the systematic, mandatory integration of Planetary Health into curricula remains elusive.

Secondly, as argued by Malmqvist and Oudin, integrating Planetary Health into medical education requires a transdisciplinary approach, involving expertise beyond traditional clinical sciences (Malmqvist and Oudin). Okatch et al. echoed this sentiment in their collaborative model in which faculty and students from diverse health professions and institutions co-developed courses on climate and health. These models illustrate how interdisciplinary education can enrich learning, foster broader systems thinking, and prepare health professionals for the complexity of environmental health challenges (Okatch et al.). Student leadership and innovation have also been identified as powerful forces for changing curricula. Pais Rodrigues et al. described a suite of 14 modular learning resources co-created by students and educators, offering flexible, accessible content grounded in authentic, learner-centered principles. This initiative demonstrates that students are not merely passive recipients of knowledge but critical agents in shaping educational responses to our Planetary Health challenges (Pais Rodrigues et al.).

The psychological burden of experiencing the triple planetary crisis must, however, be acknowledged. The studies included in this Research Topic also focus on the emotional dimensions of Planetary Health education, highlighting that, along with learner wellbeing, which must be supported through educational approaches that foster autonomy, relatedness, and competence (Pais Rodrigues et al.; Lokmic-Tomkins et al.; Grieco et al.), the emotional impact of the crisis on the population with which healthcare professional workers will be dealing must be realized. If these reforms are not included in curricula, there is a risk of disengagement.

Together, these articles invite broader debate and reflection on the evolving identity of health professionals. Healthcare professionals can no longer just be regarded as caregivers but also as advocates for a healthy planet (Pais Rodrigues et al.; Lokmic-Tomkins et al.; Ihsan et al.). The evolution of healthcare professionals' traditional roles from clinical caregiving to caring for the planet needs to be recognized and ecological consciousness, sustainability and advocacy must be integrated into professional identities (Grieco et al.; Malmqvist and Oudin; Jochem et al.).

As we delve deeper in the works in this Research Topic, questions arise that serve as food for thought for institutions and healthcare professional educators. First, how do we “center” Planetary Health education into HPE? Second, what policies and structures are needed to promote interdisciplinary collaboration across institutions, governments and the public? Third, how can we present “Planetary Health” as not just informing but inspiring awareness and health reforms in the face of overwhelming climate change, biodiversity loss and pollution?

In conclusion, this Research Topic presents a substantive and timely compilation of scholarly work examining how healthcare professions' education has evolved in response to the triple planetary crisis. The theoretical frameworks and empirical findings provide significant intellectual momentum for the advancement of Planetary Health education paradigms. These contributions collectively articulate the imperative of preparing healthcare professionals who are not only clinically competent but also have the requisite knowledge and advocacy skills necessary to address the complex interconnections between human health and ecological systems. The interdisciplinary perspectives offered in these articles establish a foundation for curricular transformation that acknowledges the inextricable relationship between planetary boundaries and health outcomes, thereby enriching both pedagogical approaches and professional identity formation in contemporary healthcare professions' education.

## References

[1] Abbasi K Ali P Barbour V Benfield T Bibbins-Domingo K Hancocks S. Time to treat the climate and nature crisis as one indivisible global health emergency. BMJ. (2023) 383:2355. 10.1136/bmj.p2355 PMC10793827 37929357

[2] McKimm J McLean M. Rethinking health professions' education leadership: developing ‘eco-ethical' leaders for a more sustainable world and future. Med Teach. (2020) 42:855–60. 10.1080/0142159X.2020.1748877 32286110

[3] Zyoud S Zyoud SH. One Health and Planetary Health research landscapes in the Arab world. Sci One Health. (2025) 4:100105. 10.1016/j.soh.2025.100105 40092474 PMC11910077

